# New Onset Diplopia in Patients with Nasopharyngeal Carcinoma following Concurrent Chemoradiotherapy: Clinical Features and Etiology

**DOI:** 10.1155/2015/735173

**Published:** 2015-10-19

**Authors:** Hui-Chuan Kau, Chieh-Chih Tsai

**Affiliations:** ^1^Department of Ophthalmology, Koo Foundation Sun Yat-Sen Cancer Center, Taipei 112, Taiwan; ^2^School of Medicine, National Yang-Ming University, Taipei 112, Taiwan; ^3^Department of Ophthalmology, Taipei Veterans General Hospital, Taipei 112, Taiwan

## Abstract

*Purpose*. To investigate the clinical features and etiology of nasopharyngeal carcinoma (NPC) patients with new onset diplopia after concurrent chemoradiotherapy. *Methods*. We retrospectively reviewed the medical records of NPC patients with new onset diplopia after concurrent chemoradiotherapy from 1998 to 2012 in a cancer center. Their clinical manifestations of ocular motor dysfunction in relation to etiology were investigated. *Results*. Twenty-three NPC patients with diplopia after concurrent chemoradiotherapy were enrolled in this study. Unilateral cranial VI palsy (91%) was the most common ocular motor dysfunction in these patients. The new onset diplopia in these patients was secondary to tumor recurrence in 12 cases (52%), radiation neuropathy in 8 cases (35%), and skull base osteoradionecrosis in 3 cases (13%). Patients with tumor recurrence and skull base osteoradionecrosis tended to present a rapid progression of the nerve palsy or severe ocular duction deficit. Patients with radiation neuropathy were often manifested by incomplete nerve palsy with insidious onset and slow progression. Patients with osteoradionecrosis were associated with poor prognosis. *Conclusions*. A new onset diplopia in NPC patients could be caused by tumor recurrence or treatment complications such as radiation neuropathy and osteoradionecrosis, and they show diverse clinical symptoms, course, and outcome.

## 1. Introduction

Nasopharyngeal carcinoma (NPC) is an epithelial malignancy with high prevalence in Southeast Asia, and radiotherapy or concurrent chemoradiotherapy (CCRT) is the mainstay treatment for this disease. The proximity of nasopharyngeal carcinoma to the skull base and cavernous sinus could cause nearby cranial nerve damage, among which cranial nerve III, IV, or VI dysfunction would result in limited ocular movement leading to diplopia. However, diplopic symptoms in NPC patients raise suspicion of not only tumor recurrence but also radiation-related cranial neuropathy. In particular, the diagnosis of radiation-induced cranial neuropathy is usually by exclusion, and a 3–6-month observation is often required to exclude tumor recurrence as the cause of nerve palsy. Therefore, how to make an early and accurate diagnosis of the etiology for NPC patients with diplopia after radiotherapy or CCRT remains a clinical challenge. In the current study, we investigated the clinical characteristics and etiology of NPC patients who presented with new onset diplopia after CCRT.

## 2. Materials and Methods

We retrospectively reviewed the medical records of patients with new onset diplopia who were previously treated with CCRT for NPC from January 1998 to December 2012 at the Koo Foundation Sun Yat-Sen Cancer Center. The CCRT regimen was the same for these patients: 7000 cGy to the main tumor with three-dimensional conformal technique before November 2003 and intensity modulation radiation technique after December 2003, followed by chemotherapy with cisplatin and 5-FU. The exclusion criteria were as follows: (1) follow-up period under 6 months, (2) diplopic symptom before CCRT completion, and (3) known persisted tumor under active treatment. When patients report a new onset diplopia, they are sent for ocular examination in the ophthalmology department and also for image study of the head and neck regions, by either magnetic resonance imaging (MRI) or computed tomography (CT). If there is any suspicious mass lesion in MRI or CT, patients would receive examination of ^18^F-2-fluoro-2-deoxyglucose positron emission tomography (FDG-PET) and nasopharyngoscopy with biopsy and microbiological culture. If the initial image study excludes suspicious mass lesion, patients would receive clinical examination regularly and follow-up image study if the diplopic symptom gets worse or other symptoms appear.

Collected data included age, gender, initial cancer stage, the latency between completion of CCRT and diplopic onset, the characteristics of ocular motor nerve palsy (type of cranial nerve, degree of ocular duction deficit, and progression of nerve palsy), the patient outcome, and the etiology of cranial nerve palsy. Ocular duction deficit was recorded on the scale described by Scott and Kraft [1]: zero (normal), −1 (to 75% full rotation), −2 (to 50% full rotation), −3 (to 25% full rotation), −4 (to midline), and −5 (inability to the midline). A complete palsy was defined as −4 or −5 duction. Rapid progression was defined as a change in scale of duction deficit ≧1 within two months. Slow progression was defined as a change in scale of duction deficit ≧1 during the follow-up period, but not significant within the initial two months. Stable condition was defined as no change of the duction deficit during the follow-up period. This study followed the tenets of the Declaration of Helsinki and was approved by the institutional review board.

## 3. Results

We enrolled 23 patients with new onset diplopia who were previously treated with CCRT for NPC in this study. There were 14 male and 9 female patients with a median age of 47 years (range 27 to 70) and a median follow-up time of 21 months (range 6 to 120 months). Three different etiologies were concluded in our series. The diagnosis of tumor recurrence is based on positive findings on MRI, CT, FDG-PET, and biopsy. The diagnosis of skull base osteoradionecrosis (ORN) is confirmed by pathological examination and positive bacterial culture of the tissues from nasopharyngoscopy. If the results of MRI, CT, FDG-PET, and nasopharyngoscopy are all negative, radiation neuropathy is diagnosed which requires a regular follow-up of at least 6 months to exclude tumor recurrence as the cause of nerve palsy. In our series, the new onset diplopia was secondary to tumor recurrence in 12 cases (52%), skull base ORN in 3 cases (13%), and radiation neuropathy in 8 cases (35%).  Table 1 shows their clinical manifestations and outcome according to the etiologies, respectively. The median latency between CCRT completion and diplopic onset was 44 months in tumor recurrence group, 48 months in ORN, and 70 months in the radiation neuropathy group (Table 2). Most patients presented with unilateral VI palsy (91%). Seven patients (30%) initially presented complete nerve palsy, in which 4 cases were caused by tumor recurrence and 3 cases were due to skull base ORN. Sixteen patients (70%) initially presented incomplete palsy with −1 or −2 ocular duction deficit, in which 8 cases had tumor recurrence and 8 cases were diagnosed as radiation neuropathy. The former 8 cases with tumor recurrence showed rapid progression of the nerve palsy, with half of them deteriorated to complete palsy. The latter 8 cases with radiation neuropathy showed either stable condition (5 cases) or slow progression (3 cases) of their nerve palsy.

Four of the 12 patients in the tumor recurrence group died during the follow-up (Table 1). The cause of death included tumor bleeding, tumor invasion of the central nervous system, and pneumonia. The 3 patients in the ORN group were all dead during the follow-up. They died of carotid artery rupture, sepsis, and liver failure, respectively. All the 8 patients in the radiation neuropathy group were alive during the follow-up.

## 4. Discussion

We demonstrated in this study that new onset diplopia in posttreated NPC patients could be caused by tumor recurrence or radiation-induced complications. Furthermore, they presented with distinct clinical characteristics, course, and outcome.

Early diagnosis of recurrent NPC is a clinical challenge. The soft tissue change after radiotherapy, such as edema, fibrosis, scarring, and loss of tissue planes, may interfere with the detection of recurrent tumor. Cranial nerves III, IV, and VI palsy as the first symptom of NPC recurrence are common with an incidence rate of 20% to 38% [2, 3]. Our current study revealed that the majority of the new onset diplopia in posttreated NPC patients was a result of tumor recurrence (52%). Patients in this group tended to have rapid progression of the nerve palsy and severe ocular duction deficit. Most of them had complete nerve palsy either as the initial presentation or during the follow-up. In a case series of 337 patients with recurrent NPC [4], Li et al. demonstrated that the common sites of tumor recurrence were in those regions not directly adjacent to the nasopharynx, for example, skull base, cavernous sinus, paranasal sinus, and orbital apex. They proposed that the radiation dose to these regions is usually low or none, so the tumor cells of subclinical lesions could survive and lead to tumor recurrence in these regions. The skull base and cavernous sinus are important aisles of multiple cranial nerves, including nerves III, IV, and VI. The limited space in these regions, coupled with the growth of recurrent tumor, probably explains the acute onset, rapid progression, and worse severity of the cranial nerve palsy seen in our series.

Despite great improvement in radiotherapy technique, skull base ORN remains one of the most serious complications for NPC radiotherapy. An estimated 2% of head- and neck-irradiated patients are at risk of developing ORN [5]. ORN results from the radiation-induced deficient cellular turnover and collagen synthesis in a hypoxic, hypocellular, and hypovascular environment, in which tissue breakdown exceeds the repair capabilities of the irradiated tissue [6]. For the first time, we reported 3 cases of skull base ORN in posttreated NPC patients presenting diplopia as the first symptom. All these three patients are male, with a relatively older age than the other two groups. In addition, they all presented acute complete nerve palsy (cranial nerve III or VI). Two of them had persisted complete nerve palsy through the follow-up. One of them recovered from ocular motor deficit after parental antibiotics; however, he died of liver failure from liver metastasis 3 years later. It has been reported that extensive ORN accompanied by radiation brain injury or cranial nerve damage had poor prognosis [7]. Despite aggressive treatment, all of the three patients in our series died during the follow-up, and one of them died of internal carotid artery rupture due to the extensive necrosis.

The incidence of radiation-induced cranial neuropathy in NPC patients was estimated to range from 1% to 5% [8, 9]. Adjuvant chemotherapy could result in increased risk of radiation neuropathy [10]. Lower cranial nerves were found to be more vulnerable. Upper cranial nerve palsy was seldom addressed in previous studies [11]. Besides, the severity and characteristic of radiation-induced upper cranial nerve palsy had never been reported in the literatures. Among the upper cranial neuropathy, the VI nerve palsy is relatively common. The vulnerability of the VI nerve is probably due to its small size [12] and its location near the skull base. In our study, all the patients in the radiation neuropathy group presented incomplete VI nerve palsy. As compared to the tumor recurrence and the ORN groups, the patients in this group had a longer latency between CCRT completion and diplopic onset, and they presented the neuropathy with an insidious onset and either slow progression or stable condition. Similar clinical presentations were also seen in radiation-induced brachial plexopathy. Harper et al. had analyzed the distinction between neoplastic and radiation-induced brachial plexopathy [13]. They found that 60% of patients in radiation neuropathy group reported little or no change in symptoms.

Accurate diagnosis of the etiology for new onset diplopia in posttreated NPC patients is difficult on occasion. MRI, CT, PET, nasopharyngoscopy, and biopsy are useful diagnostic tools to differentiate tumor recurrence from treatment sequelae. However, these examinations are either expensive or invasive, and there is limitation in all of them. For patients who have received radiotherapy, CT and MRI may have low sensitivity and specificity for distinguishing the recurrent skull base tumor from the postradiated soft tissue change [14–16]. FDG-PET is more sensitive in detecting tumor recurrence, especially for those who have inconclusive CT/MRI findings, but it is more expensive and may show false-positive in patients with ORN [17]. The final confirmation falls on pathologic examinations; however, it is often difficult to get a tissue proof from a doubtful skull base lesion. Therefore, a long-term follow-up, repeated image studies, and probably repeated biopsies are sometimes required to get a correct diagnosis. In this study, we found that the clinical presentation of ocular motor nerve palsy varied in different etiologies. Patients with recurrent tumor and skull base ORN seemed to have a rapid progression of the nerve palsy and severe ocular duction deficit. Patients with radiation neuropathy usually presented insidious onset with either slow progression or stable condition of their nerve palsy. These clinical variations cannot replace the role of image study in detecting tumor recurrence. However, the follow-up records of these clinical manifestations might give a clue to the etiology of nerve palsy when image study and nasopharyngoscopy cannot provide a definite diagnosis.

In conclusion, a new onset diplopia in posttreated NPC patients could be secondary to tumor recurrence or treatment complications. They may show diverse clinical symptoms, course, and outcome. Detailed clinical history, close observation, and follow-up of the progression in cranial neuropathy, combined with image studies and nasopharyngoscopy with biopsy, are keys to make an early and correct diagnosis for these patients.

## Figures and Tables

**Table 1:** Clinical characteristics and outcome in NPC patients with new onset diplopia in relation to etiology.

	Tumor recurrence *n* = 12 (52%)	Skull base ORN *n* = 3 (13%)	Radiation neuropathy *n* = 8 (35%)
Mean age (range)	44 (27–59)	61 (47–70)	46 (40–63)
Gender			
Male	6	3	5
Female	6	0	3
Initial cancer stage			
I	0	0	0
II	1	0	0
III	4	0	4
IV	7	3	4
Outcome			
Alive	8	0	8
Died	4	3	0

*n* = number of patients; ORN = osteoradionecrosis.

**Table 2:** Clinical manifestations of NPC patients with new onset diplopia in relation to different etiologies.

	Tumor recurrence *n* = 12 (52%)	Skull base ORN *n* = 3 (13%)	Radiation neuropathy *n* = 8 (35%)
Latency between CCRT completion and diplopic onset (range, months)	44 (16–60)	48 (7–96)	70 (40–144)

Involvement of cranial nerve			
Unilateral nerve III	0	1	0
Unilateral nerve VI	11	2	8
Bilateral nerve VI	1	0	0

Severity of ocular duction deficit at diagnosis	Complete palsy: 4 Incomplete palsy: 8	Complete palsy: 3 Incomplete palsy: 0	Complete palsy: 0 Incomplete palsy: 8

Progression of ocular duction deficit	Persisted complete palsy: 4 Rapid progression: 8 Slow progression: 0 Stable condition: 0 Recovery: 0	Persisted complete palsy: 2 Rapid progression: 0 Slow progression: 0 Stable condition: 0 Recovery: 1	Persisted complete palsy: 0 Rapid progression: 0 Slow progression: 3 Stable condition: 5 Recovery: 0

*n* = number of patients; ORN = osteoradionecrosis.
